# Loss to Followup: A Major Challenge to Successful Implementation of Prevention of Mother-to-Child Transmission of HIV-1 Programs in Sub-Saharan Africa

**DOI:** 10.5402/2012/589817

**Published:** 2012-07-31

**Authors:** Fatch W. Kalembo, Maggie Zgambo

**Affiliations:** ^1^Maternal and Child Health Department, Tongji Medical College, Huazhong University of Science and Technology, Hang Kong Lu, Wuhan 430030, China; ^2^Faculty of Health Sciences, Mzuzu University, Mzuzu, Malawi; ^3^University of North Carolina Project, Tidziwe Centre, Private Bag A-104, Lilongwe, Malawi

## Abstract

*Purpose*. The purpose of this paper was to explore how loss to followup (LFTU) has affected the successful implementation of prevention of mother to child transmission of HIV-1 (PMTCT) programs in sub-Saharan Africa. *Methods*. We conducted an electronic search from the following databases PubMed, ScienceDirect, Directory of Open Access Journals (DOAJs), and PyscINFO. Additional searches were made in WHO, UNAIDS, UNICEF, Google, and Google scholar websites for (1) peer-reviewed published research, (2) scientific and technical reports, and (3) papers presented on scientific conferences. *Results*. A total of 678 articles, published from 1990 to 2011, were retrieved. Only 44 articles met our inclusion criteria and were included in the study. The rates of LTFU of mother-child pairs ranged from 19% to 89.4 in the reviewed articles. Health facility factors, fear of HIV-1 test, stigma and discrimination, home deliveries and socioeconomic factors were identified as reasons for LTFU. *Conclusion*. There is a great loss of mother-child pairs to follow up in PMTCT programs in sub-Saharan Africa. There is need for more research studies to develop public health models of care that can help to improve followup of mother-child pairs in PMTCT programs in Sub-Saharan Africa.

## 1. Introduction

In sub-Saharan Africa an estimated 60% of people living with HIV-1 are women mostly in reproductive age group. Each year approximately 1.4 million HIV-1 positive women become pregnant. Among antenatal clients in sub-Saharan Africa the proportion of women living with HIV-1 ranges from 5% to as high as 30%, and vertical transmission is the main cause of infection among children [1]. Transmission of HIV-1 from mother to child can take place during pregnancy, labor, and delivery as well as after birth via breastfeeding especially in mixed feeding. The risk of transmission varies at different stages with the risk during pregnancy ranging from 5–10%, 10–20% during labor and delivery, and 10–20% through mixed infant feeding. It is estimated that in the absence of any intervention to prevent mother-to-child transmission (MTCT) ranges from 15–45%. This rate can be reduced to levels below 5% with effective interventions [2].

Prevention of mother-to-child transmission of HIV-1 program consists of a range of interventions, including improved antenatal services, opt-out HIV-1 counseling and testing for pregnant women, antiretroviral drug prophylaxis for HIV-1 positive pregnant women and newborns, referral to support groups, and counseling on options for safer infant feeding practices. A comprehensive PMTCT program also includes continued followup and treatment for HIV-1 positive mothers and their children, especially for the first 18 months of the child's life [1, 2].

### 1.1. Importance of Follow-Up Care in PMTCT Program

The literature shows that high attrition within PMTCT programs could be more of LTFU than of mortality. Cumulative losses in sub-Saharan African PMTCT programs are estimated to range from 20–28% during antenatal care, up to 70% at four months postpartum and close to 81% at six months after birth [3–5]. Many countries are moving towards national coverage of services for PMTCT; however, most children born to women with HIV-1 are not being systematically monitored and followed up during the postpartum period and are thus missing out on life-saving services. The followup of known HIV-1-exposed children is not only necessary to identify infants with HIV-1 and to ensure the timely initiation of treatment and care, but to also avoid postpartum HIV-1 transmission and improve overall infant health outcomes. The process of ensuring that all exposed infants and children suspected of being infected with HIV-1 receive an HIV-1 test, and if infected, receive care and treatment, provides an important opportunity for health systems to deliver comprehensive interventions for women and children [6]. One study showed that infants and children started on antiretroviral therapy (ART) when they were already severely immunodeficient never regained normal levels of immune functioning even after five years on treatment [7]. Another study showed that such infants and children are more likely to die than those children who received treatment at an earlier stage [8]. Very few children under the age of one are currently being diagnosed and subsequently receiving treatment.

Followup in PMTCT also ensures administration of a short-course antiretroviral treatment to those identified as HIV-1 positive and their exposed infants [9], provision of continuous posttest counseling and support for exclusive breastfeeding for 6months, Continuous followup of mother-child pairs through routine health services including provision of cotrimoxazole prophylaxis for opportunistic infections for mother and baby [10] and referral to community-based psychosocial support and home-based care services [11]. In June 2011, the United Nations launched theglobal plan towards the elimination of new HIV-1 infections among children by 2015 and keeping their mothers alive. The plan focuses on reaching pregnant women living with HIV-1 and their children from the time of pregnancy until the mothers stop breastfeeding [12]. Followup of PMTCT is a key factor in realizing this goal since it ensures retention of PMTCT clients and adherence to PMTCT interventions. The quality and effectiveness of PMTCT services should be assessed on the basis of the number of mother-child pairs who are receiving consistent followup and antiretroviral treatment, and the number of confirmed HIV-1-negative children born to HIV-1-positive women [13].

### 1.2. Review Objectives

The main goal of the review was to establish how loss to followup is hindering the success of PMTCT program in sub-Saharan Africa. Specific objectives of the study were to (i) determine rates of loss to followup in PMTCT; (ii) to explore the reasons for LTFU in PMTCT program; (iii) to establish the workable strategies to improve followup of clients in PMTCT program.

### 1.3. How Different Is This Review from Other Reviews?

This review is different from other reviews conducted on LTFU because of the following reasons: (i) it focuses on sub-Saharan Africa alone unlike other reviews [14, 15], which included countries from other parts of the world; (ii) the review tackles the problem of loss to followup in PMTCT programs in a comprehensive way, it includes rates of LTFU, reasons for LTFU, and strategies to improve followup of clients in PMTCT program, while other reviews [16, 17] only covered a component of LTFU, for instance Fraser et al. conducted a review study on information systems as a strategy to loss to followup, while Reithinger et al. reviewed monitoring and evaluation of programmes as a strategy to prevent mother-to-child transmission of HIV-1 in Africa. Finally, this review tackles LTFU in PMTCT of mother-child pairs, while other reviews for example Rosen and Fox, Rosen et al. reviewed retention of HIV-1-positive-adult patients in antiretroviral therapy (ART) in sub-Saharan Africa [18, 19].

## 2. Methods

### 2.1. Search Strategy

A search strategy was designed to identify publications which described LTFU in PMTCT in sub-Saharan Africa. The search was conducted from November 2011 to January 2012. We conducted an electronic search from the following databases PubMed, ScienceDirect, Directory of Open Access Journals (DOAJs), and PyscINFO. Additional searches were made in WHO, UNAIDS, UNICEF, Google, and Google scholar websites for (i) peer-reviewed published papers of studies conducted in sub-Saharan Africa; (ii) scientific and technical reports; (iii) papers presented on scientific conferences. The following search terms were used “PMTCT in sub-Saharan Africa,” “LTFU in PMTCT program,” “adherence in PMTCT program,” “retention of clients in PMTCT,” and “reasons for LTFU” strategies to improve followup in PMTCT program.”

### 2.2. Inclusion/Exclusion Criteria

Articles were included in the review if (1) they were published in English; (2) they covered any of our objectives; (3) they were published from 1990 to 2011; (4) they were conducted in sub-Saharan Africa; (5) they covered LTFU in PMTCT program (6) they involved mother-child pairs as PMTCT clients; (7) they used any type of research design.

### 2.3. Data Extraction

Included articles were studied for relevance and content. Data was extracted under the following areas: author, year of publication, country of study, study population, study objectives, research methods and interventions. The main findings of each study were summarized. 

### 2.4. Data Analysis

The data for analysis and synthesis were the study methods, findings, discussion, and conclusion. Following thematic analysis method, FK and MZ analyzed these data to identify consistent themes across the studies. Most prominent themes, explanation, and the relationship between them were identified for authors and clustered into main themes; thereafter an independent researcher verified the results of thematic analysis and made critical comments to improve the analysis. We defined “loss to followup” as 60 days or more late for schedule, consultation or medication pickup in PMTCT program. But we also accepted each study's own criteria for determining LTFU in PMTCT program. Many studies used more or less stringent definitions ranging from 1 to 6 months late for a scheduled consultation or medication pickup. Thematic analysis was used as the basis for explanation of loss to followup. We estimated the range of rate to LTFU in the studies, and we presented the results on rates to LTFU in a table.

## 3. Results

A total of 678 citations were retrieved, and after scanning titles 592 were removed because they did not address any of our objectives and inclusion criteria. The remaining 86 abstracts were read for detailed assessment, 52 articles were retrieved and subjected to detailed assessments, and 8 of these were removed because they tackled ART for adults only and some were done outside sub-Saharan Africa. The remaining 44 articles were subjected to full data extraction and thematic analysis. Articles reviewed were drawn from 11 sub-Saharan countries (South Africa, Malawi, Zambia, Zimbabwe, Kenya, Ethiopia, Mali, Kenya, Rwanda, Ivory Coast, and Uganda). After thematic analysis, the three most significant LTFU themes which were in line with our objectives and inclusion criteria were included in the study. These were (1) rates of LTFU in PMTCT in sub-Saharan Africa; (2) reasons for LTFU; (3) strategies to improve followup in PMTCT services. 

### 3.1. Rates of LTFU in PMTCT Programs in Sub-Saharan Africa

Eighteen studies were identified on rates of LTFU in sub-Saharan Africa: 4 in Malawi [20–23], 3 South Africa [5, 24, 25], 3 in Zimbabwe [11, 26, 27], 1 in Zambia [28], 1 in Ivory Coast [29], 1 in Mali [30], 3 in Ethiopia [18, 31, 32], and 2 in Kenya [33, 34]. The LTFU of mother-child pairs ranged from 19% to 89.4 in the eight countries.  Table 1 shows a summary of studies on LTFU rates in PMTCT in sub-Saharan Africa.

### 3.2. Reasons for Loss to Followup

Several factors were identified by studies as reasons for LTFU in PMTCT in sub-Saharan Africa. These included health facility factors, fear of HIV-1 test, stigma and discrimination, home deliveries, and socioeconomic factors.

#### 3.2.1. Health Facility Factors

Studies conducted In Uganda and Kenya revealed that shortage of PMTCT staff, shortages and interrupted supplies of materials, shortage of space for counseling were some of the reasons leading to loss of clients in PMTCT program. The constraints led to long waiting periods for posttest counseling, and some women left without getting their HIV-1 test results. The constraints also compromised privacy and confidentiality of mothers [30, 35]. Similar findings were also found in Kenya where 92% of respondents lacked privacy in counseling rooms, as indicated by the presence of more than 2 people in the room [36]. A study in South Africa also found that clients had inadequate information on PMTCT services, given that they could not recall the information communicated to them during counseling. Clients only made use of counseling services once during their first visit and not on subsequent visits irrespective of HIV-1 status, suggesting limited rapport between providers and clients. Experiences of those with HIV-1 positive results confirmed privacy and confidentiality were inadequate, as other clients knew the HIV-1 results of their colleagues. Findings indicated that 68% of the participants received less than 5 minutes of posttest counseling, 21% had 5–10 minutes, and only 10.7% had more than 10 minutes of posttest counseling [37]. Similar findings were found in a study conducted in Malawi where antenatal mothers thought that they were inadequately prepared to undergo HIV-1 testing. Positive mothers also thought that PMTCT had no benefit for them since ART was seen as not a part of PMTCT program. Mothers also complained of delays in getting service at ANC [38]. A study conducted in Ivory Coast revealed that mothers were afraid of being scolded at by health staff and that health workers were not attending to them when they had come for follow-up visits [39]. In Ethiopia, a study revealed that poor monitoring of PMTCT services by health workers was one of the reasons to poor followups in PMTCT program because health facilities did not have registered information on HIV-1-positive mothers who enrolled in PMTCT but failed to return for follow-up care [13].

#### 3.2.2. Fear of HIV-1 Test, Stigma and Discrimination

Studies in Malawi and Mali indicated that fear of HIV-1 test, stigma and disclosure of HIV-1 status were main reasons for LTFU in PMTCT [30, 38, 39]. A study in Zambia revealed that women feared the reactions of a partner or husband. They feared losing a husband and believed that a woman's infection and pregnancy would spark off a chain of deaths after delivery with the baby, herself and then her husband's dying. Women also feared the response of their families, believing that they will be ignored, isolated, and openly disgraced and blamed [40].

#### 3.2.3. Home Deliveries

Accessible care during labor and delivery can facilitate successful PMTCT programming. Studies conducted in Malawi and Zimbabwe showed that many existing programs suffered from high attrition rates and incomplete PMTCT followup due to the fact that many women, especially in rural areas, delivered at home rather than at a health facility [11, 20]. A study conducted in Ethiopia revealed that only 16% of births were attended by skilled personnel while in Uganda a study revealed that 71 (95.9%) of HIV-1-positive women did not return for an institutional delivery [13, 35]. Cultural influences, poor socioeconomic status, and fear of the stigma associated with an HIV-1-positive status are factors that influence choice of delivery location [13].

#### 3.2.4. Socioeconomic Factor

Understanding the socioeconomic factors that affect the ability of communities to comply with PMTCT program will assist resource-poor countries in devising strategies to achieve followup of HIV-1 exposed infant [3]. The studies conducted in South Africa and Malawi showed that socioeconomic factors such as poverty, geographical relocation, and a lack of paternal support may affect the capacity of families to comply with the PMTCT follow-up program [3, 35]. The early loss to followup may be related to the hospital's user fees or availability of free maternal and child services in the public sector in South Africa [24]. A meta-analysis conducted in sub-Saharan Africa reported that rates and barriers to disclosure of HIV-1 results amongst women varied from 16.7% to 86% and that between 3.5% and 14.6% of women reported experiencing a violent reaction from a partner following disclosure [41]. The studies conducted in Ivory Coast, Rwanda, and Zimbabwe indicated that the largest obstacle to PMTCT program was traveling long distances to health centers [39, 42, 43]. In Kenya, lack of maternal secondary education was associated with mother-baby pair nonadherence to nevirapine (NVP) [34].

### 3.3. Strategies to Improve Mother-Child Pairs Followup in PMTCT Program

LTFU is affecting the success of PMTCT in sub-Saharan Africa. Urgent strategies are therefore required to address the high rates of LTFU in PMTCT services. Studies have proposed several strategies to address LTFU. Some of the strategies include psychosocial support, family-focused approach, home visits, monitoring and evaluation, and health information systems.

#### 3.3.1. Psychosocial Support

Psychosocial support from peers helps women adhere to PMTCT program recommendations. A good example is Mothers2mothers (m2m), a clinic-based, peer-support program that provides education and psychosocial support to HIV-1-positive pregnant women and new mothers in sub-Saharan Africa. Mothers2mothers (m2m) employs HIV-1-positive mothers as peer educators and care providers in clinical health facilities to enhance the quality and effectiveness of prevention of mother-to-child-transmission (PMTCT) services. It helps women access existing PMTCT services and followups with mothers and infants after delivery. An evaluation of this program found that women participating in m2m program were significantly more likely to reveal their HIV-1 status to at least one person; receive CD4 testing during pregnancy; receive nevirapine for themselves and their infants; practice an exclusive method of infant feeding (in most cases, exclusive formula feeding) [44]. Male partner and community involvement have been found to provide psychosocial support and eventually improving retention of clients in PMTCT program [22].

#### 3.3.2. Family-Focused Approach

The family-focused approach is a distinct model of HIV-1 care which was pioneered by the MTCT-Plus Initiative at the International Center for AIDS Care and Treatment Programs (ICAP). The MTCT-Plus model of care was established to address the long-term care and treatment needs of women identified as HIV-1-infected in prevention of mother-to-child transmission (PMTCT) programs. In this model, the pregnant or postpartum HIV-1 woman is the pivotal person who serves as the guide steering her family and household members to access HIV-1 care and treatment services. The approach is distinguished by the attention to the needs of both adults and children and to the provision of comprehensive prevention and care services for all family members [45, 46].

#### 3.3.3. Home Visits

Home visits provided by health and peer workers upon enrolment to PMTCT program as well as follow-up visits if needed in case of nonadherence to scheduled visits or in case of social difficulties is helping to reduce loss to followup in PMTCT programs. Home visits include counseling and education to the families on importance of following PMTCT protocols [47]. 

#### 3.3.4. Monitoring and Evaluation

Monitoring and evaluation of PMTCT services involve developing comprehensive medical record system to collect individual patient information and to support patient care and follow-up care. Program implementation and outcomes are routinely followed including number of individuals enrolled, percentage of eligible individuals receiving ART or prophylaxis, program discontinuation (death, loss to followup, and patient withdrawal), determination of infant HIV-1 status, and CD4 cell count [48, 49].

#### 3.3.5. Information Systems

Malawi and Rwanda developed an information system using an innovative touch screen interface and HIV-1-electronic medical record. A monitoring and evaluation team uses data collected by the system to identify clients requiring follow-up care [16]. In Rwanda and South Africa several projects have used cell phones to assist in patients followup, encourage patients' compliance with treatment, and to provide medical access to medical data such as laboratory tests [50]. This technology is at an early stage in development and evaluation.

## 4. Discussion

The findings of our review revealed that rates of LTFU of mother-child pairs ranged from 19% to 89.4% in the reviewed articles. Health facility factors, fear of HIV-1 test, stigma and discrimination, home deliveries and socioeconomic factors were identified as reasons for LTFU. Strategies which are being used in some of the countries in sub-Saharan Africa to improve LTFU in PMTCT include psychosocial support, family-focused approach, home visits, monitoring and evaluation, and health information systems.

Based on the results, it can be seen that a large proportion of mother-child pairs who were registered in PMTCT programs were lost to followup. HIV-1 status of these children is therefore unknown. This implies that it is impossible to draw any conclusions on the impact or effectiveness of the program from this data. The high loss to follow-up rate also means that HIV-1-infected mothers and their exposed infants did not receive routine medical care. This calls for tailored follow-up services integrated into existing maternal and child health programs with a clear sense of ownership and accountability from staff involved in the care. The missed opportunities in infant diagnosis can also delay HIV-1-positive infants from accessing timely treatment, which is detrimental to their survival [51]. Similar findings were found in studies conducted in Europe where HIV-1-infected children were identified in a group of women who failed to complete follow-up care in PMTCT program, while their counterpart who completed follow-up care did not register any child born with HIV-1 [52, 53]. On the other hand, the results of our review are different from the findings of a study conducted in England where loss to followup among HIV-1 pregnant mothers in PMTCT was 3% and the rate of MTCT of was 2% [54]. The results of the study in England are comparable to those reported in clinical trials in developed countries such as USA where all women received ART and delivered by caesarean section [55]. Therefore, creating integrated strategies to contain the necessary procedures pertinent to HIV-1-positive women and their HIV-1-exposed infant followup at one single point within the existing under-five health clinic could be a way forward for a successful PMTCT program.

The results of the review also indicate that health facility related factors contribute a lot to high rates of LTFU in PMTCT. The results indicate the importance of positive demeanour of the staff and clear explanations of the program's procedures, for example, women who cannot complete follow-up visits need to know that they will be welcomed, not scolded, for returning again. When women return, staff members must be available to meet them. Training for program staff may be necessary in interaction skills, for instance, Thailand reported success in PMTCT services by providing training and retraining for staff on a periodic basis; enhancing counseling skills of staff; encouraging open communication and clear delineation of tasks and responsibilities [56]. A related study in Ukraine also found similar findings [57].

The results of the review have shown that home visit can play an important role in improving followup of PMTCT clients. A study in rural Bangladesh found that home visits reduce rates of LTFU in PMTCT by lowering or eliminating barriers such as transportation, financial strain, conflicts with work, and other factors that limit center-based participation [58]. Based upon the results found in rural Bangladesh, it is likely that home visits will be more successful in sub-Saharan Africa as well. 

 The findings on home deliveries suggest that satellite clinics need to provide or facilitate access to skilled birth attendants trained in both prenatal and intrapartum care for HIV-1-positive women and their infants. Adherence can be promoted through additional home visits by community health workers, who might both encourage delivery in health facilities and decrease the fear of stigma that may prevent women from delivering in designated sites. For instance, Partners in Health has demonstrated a successful model of promoting adherence to HIV-1 medications by using paid community health workers who visit each patient at home (or a location of her choice) ensuring treatment adherence through directly observed therapy, as well as including referral for HIV-1 testing with regular clinic visits, subsidizing the transportation costs, and providing medications free of charge [59].

Maternal education enhances communication between the mother and healthcare providers and also improves retention of provided information, leading to better implementation of recommended interventions. Education also empowers the woman to have autonomy in making important decisions without relying on other people [60, 61]. It is therefore important for policy makers in sub-Saharan countries to formulate policies that promote education of girls and women.

Barriers to retention of clients in PMTCT program such as poverty, HIV-1-related stigma, gender differentials and inequality, and potential for violence were also reported in studies conducted in Asia, England, and USA [62–65]. Other important PMTCT barriers to consider in planning for strategies to reduce high LTFU include the local health infrastructure, issues of transport and access, human resources availability to implement the program, necessary training of program personnel, and adequate supervision and followup. 

Drawing evidence from the results of the review, community involvement in PMTCT programs is very important for the sustainability of the programs. PMTCT clients and their families live in a community and if the community discriminates or stigmatizes them because of their HIV-1 status, it is unlikely that they can adhere to PMTCT protocols. Research shows that programs that involve community members in developing, implementing, and monitoring activities are more likely to be acceptable to the community and to have more effective outcomes [66, 67]. Conversely, failure to involve the community may not only result in a failed intervention, but may also produce unforeseen and possibly adverse effects.

### 4.1. Limitation of the Review Study

Only a few studies in sub-Saharan Africa have published information about reasons to LTFU in PMTCT. As such the review included some technical reports to substantiate on the available data. Studies eligible for inclusion in our review did not come from all countries in sub-Saharan region, some high-HIV-1-burdened countries such as Nigeria and Tanzania were left out. This may diminish the generalizability of the findings to the sub-Saharan region as a whole. Furthermore, the lack of standard definition of LTFU among studies affected our summative analysis.

## 5. Conclusion

Our review reveals that there is a great loss of mother-child pairs to followup in PMTCT in sub-Saharan Africa and the bias that comes with it in PMTCT studies is enormous and difficult to quantify. The coverage of PMTCT in sub-Saharan countries is already low, and the great loss to followup further compromises the effectiveness of PMTCT services. It can therefore be concluded that the current rate of effectiveness in PMTCT services in sub-Saharan Africa is overestimated. At this pace, it is unlikely that the goal set by UN to eliminate vertical transmission of HIV-1 by 2015 will be achieved. This calls for all PMTCT stakeholders and programmers to urgently find ways to reduce the rate of loss to followup in sub-Saharan countries. MTCT-Plus model seems to be one of the best models to improve retention of PMTCT clients in the program. Sub-Saharan countries, with great loss to followup, should try to employ this method. Further operational studies are also required to explore other public health models that can help in reducing loss to followup in sub-Saharan countries. Only few studies have been carried out to identify reasons for loss to followup, there is need for researchers to conduct further studies which are qualitative in nature in order to understand fully the reasons for loss to followup in PMTCT program. 

## 6. Recommendations

Governments in sub-Saharan Africa should formulate policies aimed at mitigating problems affecting retention of clients in PMTCT programs. Furthermore, government should allocate financial resources to build more clinics and hospitals closer to the people and to train more medical staff to meet clients' needs in PMTCT programs. In addition to this, governments in sub-Saharan Africa should introduce home visits in PMTCT programs in order to improve retention of vulnerable mother-child pairs. Utilizing trained and paid community health workers that conduct community outreach and home visits can also benefit continuity of care, follow-up care, and family involvement. It can as well help to combat some of the many barriers that women face in seeking care in PMTCT programs.

 Stakeholders from all sectors of society need to be identified and incorporated in the effort to develop a locally accepted PMTCT program. Consideration of adequate financing and sources of funding should be a central aspect of planning and program development on improving follow up in PMTCT services. The results of the review also indicated that fear of HIV-1 test, stigma and discrimination as well as low level of education were some of the reasons contributing to high rates of followup in PMTCT. This calls for integrated preventive health education not only for pregnant women and their partners, but also for the general public. At the existing sites, these can be achieved through drama performances, peer education activities at community level, and group counseling held at the antenatal clinics. This will help to raise awareness and openness in discussing HIV-1 infection and PMTCT interventions and to lessen stigma and fear surrounding HIV-1 infection. Consideration of ART access among women enrolled in PMTCT programs is also crucial. Provision of continuous training for PMTCT providers and community workers is also needed to ensure quality services in PMTCT programs. 

 Lack of HIV-1 results disclosure to male partners by HIV-1-positive pregnant mothers compounded by low male partner involvement in PMTCT program is bringing about increase in loss to followup in PMTCT program in sub-Saharan Africa._._ Strategies such as extending clinic hours accommodate men with tight work or business schedule to visit in late afternoon hours. Reducing waiting time for men or couples who visit MCH clinics in order to give chance to men to return to work and business in time. Health workers should attend ward development and other community meetings to explain the importance of male partner involvement and the need for expectant parents to be tested. PMTCT program should also integrate beliefs, values, and practices of different cultural settings so that it can attract more male partners [68, 69].

## Figures and Tables

**Table 1:** Summary of studies on rates of LTFU in PMTCT program in sub-Saharan Africa.

First author	Design	Study population	Country	Method	Results
Manzi, 2005 [20]	Cohort study	646 HIV-positive pregnant women	Malawi	Review of routine antenatal, VCT and PMTCT registers	The cumulative LTFU was 358 (55%, CI: 51–59) by the 36-week antenatal visit, 440 (68%, CI: 64–71) by delivery, 450 (70%, CI: 66–73) by the first postnatal visit, and 524 (81%, CI: 78–84) by the 6-month postnatal visit
Mirkuzie, 2010 [31]	Descriptive retrospective study	135,986 HIV-positive women.	Ethiopia	Review of PMTCT monthly reports	10.6% (896) (95% CI 9.9–11.2) of the HIV-positive women completed their followup to child HIV testing
Stringer, 2005 [28]	Surveillance study	8787 mother-infant pairs	Zambia	Mother-infant pairs surveillance	675 of 2257 (30%) seropositive mother-infant pairs received both maternal and infant dose of NVP
Sherman, 2004 [5]	Descriptive retrospective study	1234 HIV-positive mothers and their exposed infants	South Africa	Assessment of the efficacy (PMTCT) program	70% LTFU by 4 months postnatally
Perez, 2004 [11]	Cross-sectional study	2298 pregnant women	Zimbabwe	Monitoring of PMTCT Program uptake	104 (24%) mother-child pairs received nevirapine prophylaxis
Kasenga, 2007 [21]	Descriptive retrospective study	75 HIV-positive women	Malawi	Followup of HIV-positive women registered in PMTCT program	35 (47%) LTFU by delivery
Nyandiko, 2010 [33]	Retrospective cohort study	2477 HIV-exposed infants	Kenya	Comparing MTCT and infant survival rates	329 (27.4%) LTFU by 18 months of age
Kurewa, 2007 [26]	Nested case control	594 HIV-negative and 456 HIV positive mothers	Zimbabwe	Comparison between HIV-negative and HIV-positive mothers.	At 9 months the overall dropout rate was 19%
Chetty, 2011 [24]	Retrospective cohort	268 HIV-exposed infants	South Africa	Postnatal followup of data abstracted from patients records	105/260 (40.4%) infants lost to followup
Mirkuzie, 2011 [32]	Retrospective cohort study	282 HIV-positive mothers	Ethiopia	Followup of mother-infant pairs	115 (48%) mother-infant pairs LTFU by 6 weeks postnatally
Doherty, 2005 [25]	Cross-sectional study.	14340 HIV-positive women and their HIV-exposed children	South Africa	Monthly data collection on PMTCT outcomes	50% mother-child pairs LTFU by 12 months postnatally
Msellati, 2001 [29]	Routine PMTCT data collection and analysis	445 positive pregnant women	Ivory Coast	Routine collection of PMTCT data	177/445 (40%) of HIV-positive women lost to followup by the end of 6 months
van Lettow, 2011 [22]	Matched cohort study	173 HIV-infected and 214 HIV-uninfected mother-child pairs	Malawi	Followup of mother-infant pairs	28% of exposed infants were followed and tested at least once by 18–20 months of age, and only 18% of mothers followed all current PMTCT recommendations
Rosen, 2011 [18]	Cross-sectional study	113 HIV-positive pregnant women	Ethiopia	Interview and focus group discussion	71 (95.9%) of HIV-infected women were lost to followup by delivery
Kurewa, 2011 [27]	Cohort study	479 HIV-exposed infants	Zimbabwe	Followup of HIV-exposed infants	247 (51.6%) exposed infants turned up in the first year
Moth, 2005 [34]	A cross-sectional exploratory study	133 clients registered for PMTCT services	Kenya	Review of logbooks, exit interviews, indepth interviews, nonparticipant observations, and testimonies on experiences	LTFU was 11% before pretest counseling, 23.5% during HIV test, 31.5% during collection of HIV result, 53.6% during enrollment, and 80.7% at delivery
Mute, 2011 [30]	Cross-sectional survey	of 230 HIV-positive pregnant	Mali	Questionnaire and semistructure interviews	LTFU was 53% (122) of HIV-positive women
Moses, 2008 [23]	Retrospective study	20,000 HIV-positive pregnant women	Malawi	followup of mother and infant pair	35% of the infected mothers returned with their babies at 6-week postnatal followup.

## Abbreviations

PMTCT: Prevention of mother-to-child transmission of HIV
MTCT: Mother-to-child transmission of HIV
LTFU: Loss to followup
HIV: Human immunodeficiency virus
AIDS: Acquired immunodeficiency syndrome
WHO: World Health Organization
UN: United Nations
UNAIDS: United Nations Program on HIV/AIDS
ANC: Antenatal care
MCH: Maternal and child health
ART: Antiretroviral therapy.
